# Treatment and management of coenurosis by *Taenia multiceps*: field data from outbreaks in endemic regions and literature review

**DOI:** 10.1186/s13071-024-06430-2

**Published:** 2024-08-09

**Authors:** I. Abbas, C. Tamponi, G. Madau, L. Cavallo, A. Varcasia, A. Scala

**Affiliations:** 1 Department of Veterinary Medicine, University of Sassari, Sassari, Italy; 2 Parasitology Department, Faculty of Veterinary Medicine, Mansoura University, Mansoura, Egypt; 3 Veterinary Practitioner, Sardinia, Italy

**Keywords:** *Taenia multiceps*, Coenurosis, Sheep, Field work data, Treatment, Chemotherapy, Immunization

## Abstract

**Background:**

*Taenia multiceps* coenurosis is endemic in sheep from various regions worldwide. Dogs, the key hosts, shed *T. multiceps* eggs in their feces contaminating the pasture, and lambs are mostly infected during their first turnout into pastures. The disease is manifested in two forms: acute (due to the migrating oncospheres in the CNS) or chronic (due to the developing coenuri in the brain or spinal cord). Both forms are frequently accompanied by neurological symptoms.

**Methods:**

Field trials conducted in an endemic region (Sardinia, Italy) to treat replacement lambs in six sheep flocks infected with acute coenurosis are summarized in this article. The article also reviews earlier reports on various approaches developed to treat and immunize sheep against coenurosis.

**Results:**

Accurate detection of the time in which lambs become infected is crucial in deciding which treatment approach should be used. Acute disease can be successfully treated via chemotherapy. Results of field trials conducted in Sardinia revealed the efficacy of three (1-week apart) oxfendazole doses (14.15 mg/kg) in protecting apparently healthy lambs in the infected flocks from developing neurological symptoms. A single praziquantel dose (18.75 mg/kg) worked well for the same purpose and was also found significant in treating 5 of 16 clinically ill lambs in one flock. Earlier reports documented high rates of recovery (up to 100%) in clinically diseased lambs that received much higher doses (50–100 mg/kg) of praziquantel. However, chemotherapy is not preferred in chronic coenurosis since it can lead to rupture of the coenuri, giving rise to serious inflammation in the CNS. Surgical intervention is highly recommended in this case, and the pooled success rates for surgery in chronic-infected cases was estimated at 82.1% (95% CI 73.1–91.0%). However, various trials have been conducted to immunize sheep against *T. multiceps* coenurosis, and the 18k (Tm18) family of oncosphere antigens was found promising as a vaccine candidate.

**Conclusions:**

In acute coenurosis, selection of the proper anthelmintic should be done after consulting the owner for several reasons: (1) costs of the used anthelmintic: treating a small flock of 100 sheep costs around 1170 and 660 € for praziquantel and oxfendazole, respectively; (2) withdrawal time of the used anthelmintic: No time is required before consuming meat and milk from praziquantel-treated sheep, whereas meat and milk from oxfendazole-treated sheep should not be consumed for 44 and 9 days, respectively, causing additional costs for the farmers. Since no commercial vaccines have yet been developed against *T. multiceps* coenurosis in sheep, preventive measures remain the cornerstone of controlling this serious disease.

**Graphical Abstract:**

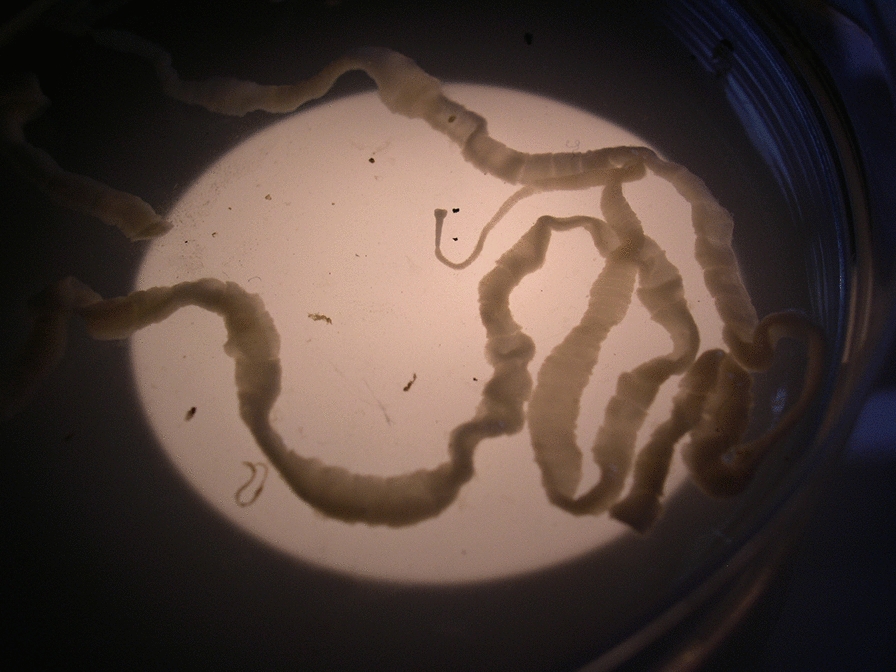

## Background

Coenurosis by *Taenia multiceps* is one of the most important parasitic diseases in small ruminants, leading to significant economic losses in their economy [1]. A few surveys conducted in Ethiopia revealed annual loss of US$ 124,821 based on occurrence of the disease in brains of apparently healthy sheep and goats, reviewed in Varcasia et al. [1]. The parasite can also cause fatal consequences in various wildlife species including deer and yak [2]. Humans can also be infected, and a few human cases of *T. multiceps* coenurosis have been reported worldwide [3]. Domestic and wild canids can serve as definitive hosts for *T. multiceps*. Although foxes, wolves and jackals have been recorded as proper hosts, shepherd dogs remain the most important host for the parasite transmission [4, 5]. The intermediate hosts include a wide variety of domestic and wild ungulates, primarily sheep and goats [6]. Infected dogs shed the gravid proglottids and/or eggs of *T. multiceps* in their feces, and eggs contaminate the environment [4]. Eggs are then ingested, in contaminated food and water, by the intermediate hosts and hatch in the intestine, giving rise to the oncospheres, which then migrate to the central nervous system (CNS) [1]. The migrating oncospheres develop to large (~ 5 cm diameter) fluid-filled coenuri within 6–8 months, leading to neurological symptoms. In sheep, cerebral coenurosis (also termed gid or sturdy) is a significant contributor to neurological disease [6]. Coenuri can also develop in other locations outside the CNS (e.g., subcutaneous tissues), particularly in goats [7].

Control of *T. multiceps* coenurosis is challenging since the parasite can circulate in various pastoral (mostly dog-sheep) and sylvatic life cycles, and its eggs can survive for long periods in the environment [8]. The permeant association between dogs and sheep herds as well as farm slaughter, unsanitary disposal of sheep offal and lack of management of coenurus-infected animals in pastures (very common in extensive breeding) contribute to the widespread occurrence of coenurosis in the endemic regions [1, 5, 9]. Sardinia is an obvious example for these regions, where the disease is known by the farmers, and the parasite is typically responsible for several neurologically diseased cases in sheep farms [10, 11]. In addition, atypical outbreak of acute coenurosis has been documented in lambs < 30 days old in a flock from Sardinia [12]. This emphasizes the potential occurrence of *T. multiceps* coenurosis in replacement lambs, giving rise to extensive mortality episodes due to acute disease. Herein, we provide field experience to treat replacement lambs in six Sardinian sheep flocks with several cases of acute coenurosis.

Although several articles and reviews have been published so far on coenurosis, a few of these articles have focused on treatment and management of this parasitic disease, which highlights the importance of summarizing, in a single contribution, all the most important effective treatment therapies and control strategies against this serious disease. The present article, therefore, reviews the literature on various treatment approaches and vaccines developed to control *T. multiceps* coenurosis.

## Methods

### Field work data

**Fig. 1:**
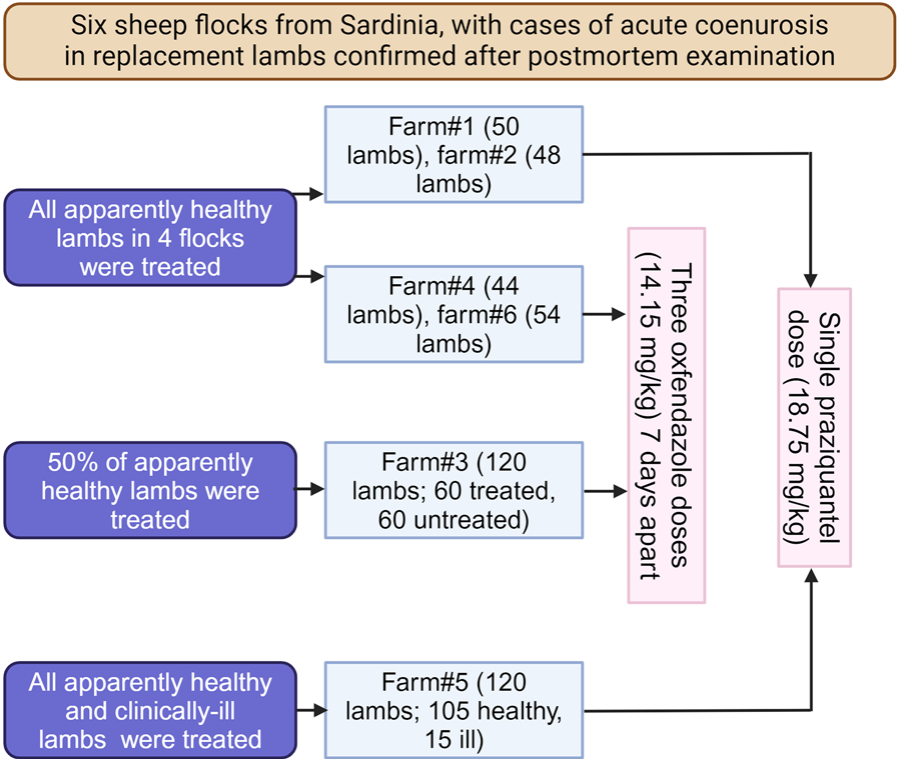
Various regimens used in the present study to treat acute coenurosis in six sheep flocks from Sardinia, Italy

From March 2020 to April 2024, six different outbreaks of coenurosis by *Taenia multiceps* occurred in six Sarda breed sheep farms in Sardinia, Italy. These farms were located in six different municipalities belonging to two provinces: Nuoro (farm #1 in Bitti and farm #2 in Oniferi) and Sassari (farm #3 in Tula, farm #4 in Pattada, farm #5 in Thiesi and farm#6 in Benetutti). Table 1 summarizes information on the six flocks, and data mentioned in this section are based on field experience of one of the authors (Madau G), who works as a local veterinary practitioner in Sardinia. The included farms contained 250–500 sheep reared for dairy purposes. Each farm contained 50–120 replacement lambs. Most of the coenurosis cases occurred in replacement lambs aged 4–6 months; however, acute infections were also observed in sheep aged 1–3 years in the Oniferi flock (farm #2). Around 18—24% of the replacement lambs displayed neurological symptoms, including separation from the flock, ataxia, lateral recumbency and head tilt. Some episodes of sudden deaths had also occurred. The disease was confirmed after necropsy of all clinically ill lambs in four flocks and a few clinically ill lambs in the remaining two flocks (#4 and 5). The infected animals had typical lesions of acute coenurosis. Hyperemia of the vessels on the brain surface and several necrotic tracks of *T. multiceps* oncospheres migrating in the CNS were observed [1]. After diagnosis, off-label therapies of commercial preparations containing either praziquantel (Neomansonil^®^, 25 mg/ml) or oxfendazole (Oxfenil^®^, 2.265 g/100 ml) were administered. Informed consents from the owners were taken orally by Madau G. The owners were informed about the disease and consequences of treating or not treating their animals. The anthelmintic preparations were given orally in different regimens; see Fig. 1. For example, animals in three flocks (#1, 2 and 5) received a single 30 ml dose of the praziquantel preparation (i.e. 562.5 mg) equivalent to 18.75 mg/kg. Animals in the remaining 3 flocks (#3, 4 and 6) received three 25 ml doses (i.e. 566.25 mg) of the oxfendazole preparation equivalent to 14.15 mg/kg 7 days apart. All apparently healthy lambs in the six flocks were treated, but in flock #3, only 50% of the apparently healthy lambs were treated, whereas the remaining 50% did not receive any treatments. Besides, clinically ill lambs in two flocks (#4 and 5) were treated. The six flocks were then carefully monitored for a long period.

**Table 1:** Field off-label treatment used in coenurosis-infected sheep reared in six different flocks in Sardinia, Italy

Farms, region	Total replacements sheep raised	Animals showing acute symptoms	Veterinary practitioner notes	Treatment	Outcomes
Farm #1, Bitti (400 head)	80	30	30 animals with neurological signs were slaughtered. The remaining 50 were treated	Praziquantel* 30 ml (750 mg; 18.75 mg/kg) per sheep in a single shot	After treatment five animals showed symptoms and were slaughtered; other animals survived
Farm #2, Oniferi (300 head)	60	12	12 animals with neurological signs were slaughtered. The remaining 48 were treated	Praziquantel* 30 ml (750 mg; 18.75 mg/kg) per sheep in a single shot	Surprisingly, infected sheep were 3 years old, and infections occurred in 1–2-year-old sheep. After treatment three animals showed symptoms and were slaughtered; other animals survived
Farm #3, Tula (500 head)	120	10	Only 50% (n = 60) of replacement sheep were treated	Oxfendazole** 25 ml (566.25 mg; 14.15 mg/kg) per sheep, 3 doses 7 days apart	Animals treated did not show any other symptoms. Almost all (90%) untreated animals died of coenurosis
Farm #4, Pattada (250 head)	50	6	All animals were treated	Oxfendazole** 25 ml (566.25 mg; 14.15 mg/kg) per sheep, 3 doses 7 days apart	Animals treated did not show any other symptoms
Farm #5, Thiesi (500 head)	120	15	All animals were treated	Praziquantel* 30 ml (750 mg; 18.75 mg/kg) per sheep in a single shot	In this farm even sheep showing clinical signs of acute gid were treated, and 6 out of 15 survived. No other cases were recorded after treatments
Farm #6, Benetutti (300 head)	60	6	6 animals with neurological signs were slaughtered, whereas 52 animals were treated	Oxfendazole** 25 ml (566.25 mg; 14.15 mg/kg) per sheep, 3 doses 7 days apart	Animals treated did not show any other symptoms

*Neomansonil^®^ oral suspension (Elanco) 25 mg/ml–withdrawal periods: meat and milk 0 days

**Oxfenil^®^ oral suspension (Virbac Srl) 2.265 g/100 ml–withdrawal periods: meat 44 days; milk 9 days (18 milkings)

### Literature review

Various databases (e.g. Scopus, PubMed, ScienceDirect and Google Scholar) were searched to collect all published data on treatment and control of *T. multiceps* infections. Various keywords were used in various combinations, including *T. multiceps*, coenurosis, sheep, goat, dogs, treatment, vaccination, immunity and control. These keywords were combined using the Boolean operators “AND” and “OR.” Full texts of the published papers were collected. Abstracts of the previously published papers that were not available in full texts were collected from the CABI database (https://www.cabidigitallibrary.org/product/ca/fulltext). Important findings of the collected studies are summarized in the tables and throughout the text. The software Open Meta (Analyst) was used to estimate the pooled success rate of the surgical treatment of coenurosis based on a 95% confidence interval [13].

## Results and discussion

Results of chemotherapeutic management of six coenurosis-infected sheep flocks in Sardinia are summarized in Table 1. The replacement lambs in these flocks received off-label doses of two anthelmintics, either praziquantel or oxfendazole. After long surveillance, no apparently healthy lambs that received the three oxfendazole doses developed any neurological symptoms. Additionally, in the case-control experiment conducted in flock #3, lambs that were given oxfendazole did not develop any neurological symptoms, whereas the majority (90%) of untreated lambs did and even died of coenurosis. On the other hand, variable results were revealed when treating lambs in three flocks with the praziquantel. A few praziquantel-treated lambs in flocks #1 (5/50) and #2 (3/48) developed neurological symptoms. This was not the case in flock #5; no neurological symptoms were observed in any apparently healthy lambs that received a single praziquantel dose. In addition, 5 of the 16 clinically ill lambs that also received a praziquantel dose were successfully treated.

These field work data illustrated the efficacy of large doses of either praziquantel or oxfendazole in protecting the replacement lambs against acute coenurosis. In addition, large praziquantel doses can treat a significant portion of the already infected lambs showing neurological symptoms. However, this field work does not provide data on the efficacy of both anthelmintics in treating chronically infected cases. In the following sections, we discuss our field work data along with results of the earlier published experiments to give a comprehensive overview on various approaches used to treat and immunize sheep against coenurosis.

### Treatment of *T. multiceps* coenurosis

Treatments of *T. multiceps* coenurosis can be performed at various stages of the disease, and the most important thing to achieve success is to determine the approximatel time when animals were infected. For example, if we had recently infected animals, such as replacement lambs with acute infection by *T. multiceps* oncospheres in early stages of migration, then it would be better to use chemotherapy (i.e. anthelmintic treatment). The timing in this case is very important because using anthelmintic treatments in animals with already vesicolized oncospheres could result in rupture of the coenuri, giving rise to serious inflammatory consequences in the CNS. However, if we are managing animals with symptoms of chronic coenurosis (with vesicolization of oncospheres and coenuri in different stages of maturation), then we can determine the exact localization of the coenuri with the help of imaging diagnostics and decide to perform a surgical intervention.

#### Chemotherapy

Earlier reports have used several anthelmintics in different regimens to treat coenurosis in naturally and experimentally infected sheep and goats (Table 2). However, it is thought that chemotherapy can only be used during the migration stages of the parasite since the fully grown coenurus can rupture after treatment, which threatens the life of the infected animal [1]. The efficacy of the used anthelmintic was evaluated based on clearance of the neurological symptoms, degenerative changes occurring in the recovered coenuri from treated animals and the ability of these coenuri to develop into adult worms when experimentally fed to dogs.

**Table 2:** Chemotherapeutic trials to treat cerebral and non-cerebral coenurosis in sheep and goats

Animal	Location of coenuri	Infection (dose)	No. treated animals	Start treatment	Medication (route of administration)	Dose mg/kg (period)	Efficacy %	Efficacy testing, time after treatment	Remarks	Reference
Lambs 5 months old	Cerebral	Exp (2400 eggs)	2	36 days	MBZ (IP)	40 (S)	0.0	Degenerative changes in coenuri, 78 days PT	MBZ is ineffective to treat cerebral coenurosis	[14]
			3		MBZ	100 (14 days)	0.0			
Lambs 4–5 months old	Cerebral	Exp (6500 eggs)	5	CL	ALB	25 (6 days)	100	Degenerate/dead coenuri, after recovery	2/5 and 1/5 of FEN and PRZ-treated groups died before necropsy. Likewise, 5/5 of control group died before necropsy. Caseated and calcified cysts were noticed in the treated groups, but most commonly in those treated with ALB or FEN-PRZ comb. Authors claimed albendazole was the most effective	[17]
			5		FEN	25 (8 days)	71.4			
			5		PRZ	100 (7 days)	85.3			
			5		FEN-PRZ Comb	****	100			
			5		Control	–	0.0			
Lambs 3–4 months old	Cerebral	Exp (5500 eggs)	7	CL	PRZ	25 (3 days)	100	Degenerated/dead coenuri, after recovery	5/7 lambs from PRZ group and 4 of ALB group died before necropsy. Dogs fed protoscolices from treated groups were not infected	[20]
			7		ALB	10 (14 days)	54.8			
			7		Control	–	2.8			
Sheep	Cerebral	Nat	2	CL	PRZ	7.5 (6 days)	NS	Clearance of neurological symptoms	One PRZ-treated animal survived, and general conditions of the remaining animals worsened. No improvement occurred in OXF-treated sheep	[24]
			6		PRZ	50 (2 days)	NS			
			2		OXF	30 (S)	0.0			
Sheep	Cerebral	Nat	NS	–	PRZ	100	NS	Degenerated cysts, transmission to dogs	Calcified coenuri in brains of treated sheep. Coenuri in untreated sheep were alive. Dogs fed on brains of treated sheep did not have tapeworms	[26]
Sheep	Cerebral	Exp (50,000 eggs)	1	15 days	PRZ	100 (5 days)		21-day PT	Clinical signs of acute coenurosis appeared 17-day PI. No improvement occurred when treated. Numerous migratory tracts were evidenced in the brain. No live coenuri were detected	[28]
		Exp (n = 11; 5000 eggs), Nat (n = 2)	2	2–11 months	PRZ	100 (5 days)	0.0	Recovery (clearance of neurological symptoms), dead coenuri, transmission to dogs, 1–> 3 months PT	Clinical symptoms disappeared. No coenuri were recovered from the brains	
			2		PRZ	100 (2 days)	100			
			4		PRZ	100 (S)	100			
			1		PRZ	50 (5)	100			
			4		PRZ	50 (S)	50.0		Variable response to treatment. Clinical improvement occurred in 2 sheep and dead coenuri were detected in their brains. One sheep responded but the clinical symptoms reappeared 3 months later One sheep did not respond and mature coenuri were detected in the brain	
Sheep	Cerebral	Exp (5500 eggs)	2	CL	PRZ	100 (5 days)	100	Non-viable/degenerated coenuri, 30-day PT	All treatments were effective. Dogs that fed on the coenuri collected after killing the included sheep, did not develop adult worms. PRZ is highly effective	[29]
			2			100 (2 days)				
			1			50 (5 days)				
Goats	Non-cerebral	Exp (3000 eggs)	7	2 months PI	ALB	10 (3 days)	90.3	Non-viable/viable coenuri, 10 days PT	Significant variation in occurrence of non-viable coenuri when goats received albendazole 2 months post infection	[32]
			6	2 months PI	ALB	10 (S)	72.7			
			6	2 months PI	ALB	20 (S)	73.9			
			12	2 months PI	Control	–	13.6			
			5	5 months PI	ALB	10 (3)	88.6			
			5	5 months PI	Control	–	84.6			
Goats	Non-cerebral	Nat	12	–	FEN	7.5	NS	–	FEN is effective in treating non-cerebral coenuri in goats	[33]
Sheep	Cerebral	Nat	20	–	FEN-PRZ Comb	10 + 3 (5 days)	84.0	Recovery (clearance of neurological symptoms)	Neurologically-diseased. No confirmed diagnosis, based on the history and clinical signs	[48]
Sheep	Cerebral	Nat	NS	–	PRZ	50–100 (3 days)	NS	Occurrence of neurological symptoms	Apparently healthy sheep from flocks with endemic coenurosis. No cases have been reported for 6 months PT	[49]
Sheep	Cerebral	Nat	61	–	PRZ	80–100	NS	Recovery (clearance of neurological symptoms)	56 sheep received treatment. Recovery occurred in 43 (70.5%), and 13 died. Intramuscular administration gave better results than the oral route	[50]
					PRZ (IM)	40–50	NS			
Lambs	Cerebral	Nat	3	CL	ALB	25 (2 doses 2 weeks apart)	0.0	Clearance of neurological symptoms	The treated lambs (*n* = 3) died despite this treatment. No new cases of the disease were observed after the initiation of control measures	[51]
Lambs	Cerebral	Exp	5	NS	FEN	0.75 –1 g	NS	Occurrence of neurological symptoms	No clinical signs appeared in any treated animal. Acute signs of the disease occurred in the controls. No coenuri were detected in brains of the treated lambs. FEN was administered prophylactically to lambs in a flock with a history of coenurosis, and symptoms of the disease did not recur	[52]
			5		PRZ	25 (6 doses 20-day interval)				

*ALB* albendazole, *FEN* fenbendazole, *PRZ* praziquantel, *MBZ* mebendazole, *OXF* oxfendazole, *NS* not stated, *Exp* experimental, *Nat* natural, *S* single, *Comb* combination, *PT* post treatment, *PI* post infection, *CL* treatment starts at appearance of clinical signs; IP, intraperitoneal; IM, intramuscular

****FEN 0.5 g/head, PRZ 100 mg/kg–6 times 20-day interval

All treatments were orally administered to animals

Various benzimidazoles were trialed, including albendazole, fenbendazole, oxfendazole and mebendazole. The latter was found ineffective in treating coenurosis [14]. Mebendazole also has a lower efficacy in treating cystic echinococcosis in humans since it has lower systemic absorption and penetration into hydatid cysts compared to the albendazole [15]. The superior efficacy of albendazole might also be related to its activity against protoscoleces [16]. In coenurosis, albendazole at a dose of 25 mg/kg for 6 consecutive days (i.e. a total dose of 150 mg/kg) was successfully used to treat neurologically diseased lambs after experimental infection [17]. Albendazole penetrates well into the CSF, which increases its efficacy [18], and this can explain the higher efficacy of albendazole than praziquantel in treating human neurocysticercosis caused by *Taenia solium* [19]. However, when the experimentally infected sheep received a lower albendazole dose of 10 mg/kg for 14 consecutive days, 50% of the coenuri displayed degenerative changes, whereas the other coenuri remained alive [20]. The efficacy of benzimidazoles, in general, correlates with sustained plasma level of the drug derived from administering effective concentrations over a prolonged time. This allows prolonged interaction between the drug and the parasite, giving rise to higher efficacy [21]. However, prolonged use of albendazole has been associated with bone marrow toxicity in sheep [22]. Notably, albendazole is frequently used to control gastrointestinal nematodes, lung worms and liver flukes that commonly infect sheep and goats in doses ranging from 5 to 10 mg/kg [23].

In our field work, oxfendazole, one of the most commonly used anthelmintics to control various parasitic infections in livestock, has been shown very effective in protecting apparently healthy lambs from developing coenurosis, when given at a cumulative dose > 1.5 g (1.698.75 g) divided over three doses. However, earlier studies scarcely reported use of this anthelmintic to treat coenurosis, and a single 30-mg/kg oxfendazole dose was not found effective in treating two neurologically diseased sheep with cerebral coenurosis (Table 2) [24]. Unfortunately, the efficacy of oxfendazole in treating lambs with clinical coenurosis was not tested in our field work.

However, praziquantel, an isoquinoline derivative, has been tested in several studies and displayed great efficacy in treating coenurosis as well as preventing early disease; see Table 2. Praziquantel has great cestocidal activity and is very safe when given orally or parenterally [25]. This anthelmintic is also widely used to treat cystic echinococcosis in humans since it can inhibit the vesicular evolution of protoscoleces and has a role in loss of viability of small cysts [26]. Praziquantel is commonly used to treat coenurosis at a dose of 50–100 mg/kg, and this dose has been described based on results of earlier experiments (Table 2). Historically, Bankov (1977) was the first to use praziquantel to treat sheep coenurosis and found that two doses of 50 mg/kg praziquantel were successful in treating acute coenurosis, reviewed in Eslami and Bazargani [27]. However, a five times higher dose (100 mg/kg for 5 days) failed to treat acute infection in a lamb that was experimentally fed 50,000 *T. multiceps* eggs [28]. This suggests that the efficacy of praziquantel at a given dose correlates with the intensity of the parasite. However, it is important to note that the dose of 50,000 eggs is huge and probably 8–10 times bigger than that of common field infections (Varcasia, personal observation).

Verster and Tustin [28, 29] did the most comprehensive trials to detect the appropriate praziquantel dose for treating sheep coenurosis. The authors used various praziquantel treatment regimens in naturally and experimentally infected sheep. Results of their experiments are detailed in Table 2. In conclusion, a single dose of 100 mg/kg was found effective. This was also evidenced when a dead coenurus (based on transmission to dogs) was revealed from a brain of sheep killed 2 days after receiving a single 100 mg/kg dose of praziquantel. Unsatisfactory results were revealed when using the single 50 mg/kg dose. No or temporary clinical improvement occurred in two of the four sheep that received this dose. Nevertheless, according to our field work, a much lower praziquantel dose (18.75 mg/kg) can protect apparently healthy lambs and can also treat some neurologically ill lambs (Table 1). On the other hand, the combination praziquantel-fenbendazole was occasionally tested in earlier reports, and the results were satisfactory, but full doses of both anthelmintics were required [17]. A combined therapy of two anthelmintics (e.g. praziquantel-albendazole) is commonly used pre- and post-surgical intervention in cystic echinococcosis. This combination can reduce the risk of disease recurrence and intraperitoneal seeding of infection, which develops from cyst rupture and spillage of the hydatid fluid [26]. In humans, praziquantel-albendazole combination has also been used as a postoperative therapy after surgical removal of *T. multiceps* coenuri from the brain of a 4-year-old girl [30]. Notably, praziquantel is the only drug used to successfully control *T. multiceps* infections in dogs [31].

Compared to cerebral coenurosis, reports on treating the non-cerebral form of the disease are few, probably because of its non-life-threatening nature. Albendazole at a dose of 10 mg/kg for 3 consecutive days significantly reduced the viability of the early 2-month developed coenuri in experimentally infected goats. However, the viability was similar to that of the controls when treating goats that had 5-month-old coenuri [32]. Fenbendazole at a dose of 7.5 mg/kg was also found effective [33] (Table 2).

Overall, some practical considerations should be considered when selecting an anthelmintic to treat coenurosis under field conditions. First, the treatment protocols should be sustainable, meaning applicable by farmers. Protocols with too many interventions are usually abandoned by farmers because they cannot follow them up. Therefore, any useful treatment with a single administration should be welcomed. Second, while the local practitioner can select any protocol to treat ruminants bred as pets, it is crucial for farmers to consider the cost-effect ratio since all replacement lambs should usually be treated, including both symptomatic and asymptomatic animals; otherwise, the untreated asymptomatic animals may develop chronic coenurosis a few months later. Cost of the therapy is also important because 3–5 times higher anthelmintic doses are occasionally used during the off-label treatments. According to Table 2, for example, treating a single animal with a single dose of 18.75 mg/kg praziquantel or three doses of 14.15 mg/kg oxfendazole results in an economic investment of 11.7 and 6.6 €, respectively. The costs of treatment in a small flock of 100 sheep are therefore 1170 and 660 €, respectively, for the two drugs. This difference is huge. However, even with the use of praziquantel, the costs of treating all replacement lambs are less than the economic loss incurred by the untreated lamb's deaths. For example, flock #3 lost 54 (90%) of the 60 untreated lambs, and each replacement lamb costs around 90 €. This results in 4860 € loss, which is much less than the 720 € loss due to praziquantel treatment of those 60 lambs.

**Fig. 2:**
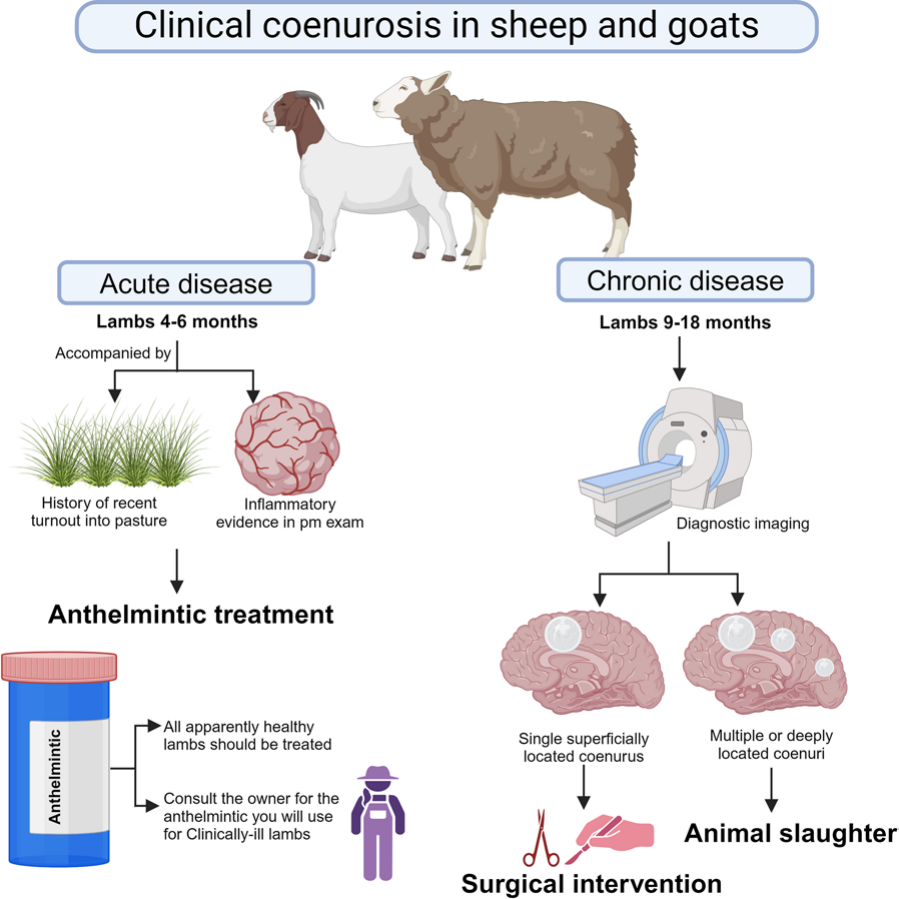
A guide suggested for veterinary practitioners to manage sheep and goat flocks with cases of cerebral coenurosis

Lastly, the other practical aspect to consider is the withdrawal time for consuming products derived from the treated animals. In praziquantel-treated animals, no time is required to consume meat and milk according to different pharmacological formulations. Praziquantel is characterized by rapid absorption, reaching the peak plasma concentration within 2 h of dosing, and around 90% of the total dose administered is excreted within 24 h [34]. Contrarily, oxfendazole can be detected in the plasma up to 144 h after oral dosing. Therefore, a long time is required before meat (44 days) and milk (9 days equivalent to 18 milking) from oxfendazole-treated animals can be consumed [35]. Therefore, in case of treatment failure, farmers can finally decide to slaughter praziquantel-treated lambs to minimize the economic loss. This is not the case in oxfendazole-treated lambs since farmers will have to destroy the carcasses, incurring additional costs (90–120 € per lamb plus the destruction costs). However, the 9 days off milking for a single oxfendazole-treated lactating ewe will result in 25 € loss since a primiparous sarda ewe can produce around 15 l in 9 days, and each liter costs around 1.70 €. A practitioner guide suggesting that veterinarians manage coenurosis-infected flocks is given in Fig. 2.

#### Surgical treatment

The surgical removal of the coenuri from brains of chronically infected sheep is frequently successful [36]. A very important step in this process is to detect the exact number of coenuri present in the brain or spinal cord and their exact localization, and this can be successfully performed using magnetic resonance irradiation (MRI). Although it has commonly been believed that coenurus infections are usually caused normally by a single cyst, Varcasia et al. [1] reported that 30% of infected animals had multiple cysts, and the number of coenuri in a single brain can be up to 12.

The surgical procedures include grasping the coenurus through a hole (~ 0.5 cm in diameter) in the skull, aspiration of the fluid inside and removal of the collapsed coenurus through gentle traction of its wall [37, 38]. Surgical procedures to aspirate the fluid without removing the collapsed coenurus were also effective [39], reporting neurological symptoms associated with increased intracranial pressure due to development of coenuri. Table 3 summarizes important findings of the earlier reports on surgical removal of coenuri from brains of infected sheep and goats. Data from these reports were combined, and recovery occurred in 898 of 1128 sheep subjected to surgery, with an estimated pooled success rate of 82.1% (95% CI 73.1–91.0%). The recovery usually occurs 1–4 weeks post-surgery. However, surgery is not commonly practiced under field conditions; it is usually restricted to economically valuable animals and requires a professional practitioner. In Italy, surgical procedures to remove brain coenurus cost the owners around 300–500 €, considering that they should also include a CT scan or MRI prior to surgery to assess the exact positions of coneuri in the SNC. In addition, this approach is not successful in cases with deeply located coenuri (e.g. in the cerebellum and brain stem) or in cases with multiple coenuri. Deaths can also occasionally occur during the surgery (see Table 3). In addition, horns of male sheep and goats constitute a significant obstacle to this treatment strategy [38].

**Table 3:** Reports on surgical treatment of cerebral coenurosis in sheep and goats

Animal	Diagnosis	No. of cases	Recovered cases (%)	Cases with no improvement	Animals died during surgery	Remarks	Reference
Sheep	MRI	3	3 (100)	0	0	These sheep were infected with 3000–7000 *Taenia multiceps* eggs collected from naturally infected foxes. Two sheep had a single coenurus. One sheep had 4 coenuri in the brain	[5]
Goat	MRI	1	1 (100)	0	0	This goat was infected with 5000 *T. multiceps* eggs collected from naturally infected foxes and developed 3 coenuri in the brain	
Dwarf goat	NE, MRI	1	1 (100)	–	–	Progressive improvement in symptoms occurred in the next 3 months PT	[36]
Goat	CL, NE	^†^52	48 (92.3)	4	0	Recovery occurred 1–4 weeks PT. The 4 cases suffered from reoccurrence of the neurological symptoms 2 months PT	[37]
Sheep	History, CL, NE	^†^*623	**517 (82.9)	***56	13	350 had immediate improvement PT, 126 improved gradually In the next 2–5 weeks PT	[38]
Sheep	NS	^†^220	141 (51.8)	NS	NS	–	[53]
Sheep	CL	^†^42	31 (73.8)	NS	NS	–	[54]
Sheep	CL, radiography	11	NS	NS	NS	Successful clinical response was observed PT	[55]
Sheep	CL, radiography	^†^7	5 (71.4)	0	2	–	[56]
Sheep	NS	^†^132	106 (80.3)	NS	NS	–	[57]
Sheep	NS	^†^17	14 (82.3)	2	1	–	[58]
Sheep	CL	20	NS	NS	NS	Long surveillance indicated that no recurrence of symptoms occurred	[59]
Goat	NE, UE	3	3 (100)	0	0	Surgery was not conducted in an additional case that had a deeply located coenurus	[60]
Goat	CL, NE	^†^35	35 (100)	0	0	–	[61]
Goat	CL	1	1 (100)	0	0	–	[62]
Goat	CL. Radiography in 1 case	5	NS	NS	NS	–	[63]
Goat	History, CL	10	NS	NS	NS	Animals improved the 2nd day PT	[64]
Ibex	CL, CT	2	2 (100)	0	0	–	[65]

*CL* clinical findings, *NE* neurological examination, *MRI* magnetic resonance imaging, *CT* computed tomography, *UE* ultrasound examination, *PT* post-treatment

^†^Included to estimate the pooled success rate of the surgical treatment

*In the remaining 37 cases, the coenuri were not localized

**36 displayed low level improvement

***Included 56 animals that displayed temporary improvement followed by relapse. After slaughtering, 2–5 coenuri were found in their brains

### Immunity against *T. multiceps*

Several factors may contribute to the development of immunity against *T. multiceps* infections in the intermediate hosts. Age-related resistance can be suggested since most cases with clinical coenurosis occurred in young animals. Sheep aged 1–3 years had three times more infections compared to sheep > 3 years old [6]. In addition, the younger the animal was, the more likely the infection would become patent [8]. Feeding on colostrum may also play a role in development of at least partial immunity against coenurosis since infections can frequently occur in the first few weeks of life [8, 12]. Herbert et al. [8] found that experimentally infected lambs that had not received colostrum during their first few days of life had more infections than those fed on colostrum.

Earlier experiments also suggested occurrence of some degree of resistance to *T. multiceps* eggs/protoscoleces in the definitive/intermediate hosts. For example, Herbert et al. [8] experimentally fed 1000–5000 viable *T. multiceps* eggs to each of 107 3–27-week-old lambs. Brain invasions were evidenced only in 49 lambs, including 37 that developed mature coenuri. The coenuri failed to develop in 12 cases. This indicates that only a small proportion of ingested *T. multiceps* eggs can develop into mature coenuri, even when the oncospheres reach the CNS. On the other hand, Willis and Herbert [9] fed five dogs 50–80 *T. multiceps* protoscoleces harvested from freshly collected coenuri. Dogs were then purged at varying times. Not all the ingested protoscoleces developed into mature worms. The reasons for this partial resistance to eggs/protoscoleces are not clear. In the intermediate hosts, many of the oncospheres migrate outside the CNS to the liver, heart, kidney, diaphragm and skeletal muscles, where they display limited development before being attacked mostly by neutrophils. These unsuccessful infections may have a role in developing some degree of protection [8].

No trials have been conducted to immunize dogs against *T. multiceps*. However, dogs can turn refractory after repeated infections 2–3 times in a short period [40]. On the other hand, various experiments have been conducted to immunize sheep and goats against *T. multiceps* coenurosis, and various types of antigens have been used, including the oncosphere antigen and recombinant protein antigens. These experiments are summarized in Table 4. Results of the experimental studies conducted by Verster and Tustin [41, 42] documented the appropriateness of oncosphere secretory antigen (OSA) as a vaccine candidate to induce protective immunity against the larval stages of *T. multiceps*, particularly when given in two doses; see Table 4. In addition, Verster and Tustin [42] found that vaccinated lambs (1–4 months old) from either OSA-vaccinated or unvaccinated mothers displayed similar levels of protection against *T. multiceps* larvae, and this protection was significantly higher than that from unvaccinated lambs of both groups. This suggests that the passive immunity transferred from vaccinated mothers to their offspring lasts for short periods, and all newborn lambs should be vaccinated in the first few days after birth. Under field conditions, OSA vaccination was applied in a sheep flock with endemic coenurosis, and no further cases were observed in the vaccinated animals. However, *T. multiceps* was detected in the dogs associated with this flock when they were sampled 2–3 times during the experiment [42].

**Table 4:** Immunization trials against *Taenia multiceps* coenurosis

Recruited animal	No. animals	Antigen used	Administration route, doses (interval)	Challenge dose (eggs)	Immunization to first challenge (days)	Challenge to necropsy (days)	*Infected animals	Remarks	Reference
Lambs 4–8 weeks old	6	TmAO	SC, 1	2000	35	138	1	Activated oncospheres provide some degree of protection. Insignificant variations between these 4 groups in number of infected lambs, those with evidence of infection or in numbers of coenuri. Prior exposure of these lambs to *T. multiceps* was suggested	[40]
	5	ThAO	SC, 1	2000	35	138	3		
	6	Cf	SC, 1	2000	35	138	2		
	6	Control	–	2000	35	138	3		
Lambs 4–8 weeks old	5	OA	IM, 1	2000	25	146	1	Significant protection in lambs vaccinated with oncosphere-derived antigens	
	5	Control	–	2000	25	146	5		
Sheep 3–7 months old	3	OSA	2 (2 weeks)	6000	14	33	0	Degenerate lesions in various organs occurred in all the controls and only in one vaccinated animal	[41]
	3	Control	–	6000	14	33	3		
Lambs 4 months old	30	OSA	2 (2 weeks)	5000	14	36–38	5	The majority of animals in the vaccinated group had degenerate lesions in the liver	
	11	Control	–	5000	14	36–38	8		
Lambs 4–6 months	9	OSA	SC, 1	5000	42	90	4^†^	2 doses 2 weeks apart are sufficient for lambs to develop complete resistance against coenurosis	
	10	OSA	SC, 2 (2 weeks)	5000	42	90	0		
	10	FD-OSA	SC, 2 (2 weeks)	5000	42	90	0		
	10	Control	–	5000	42	90	8		
Lambs 1–2 months	43	OSA	SC, 2 (4 weeks)	4600	140	81–96	2	Lambs from vaccinated mothers with 2 OSA doses 90 and 120 days of pregnancy	
	24	Control	–	4600	140	81–96	13		
Lambs 3–4 months	42	OSA	SC, 2 (4 weeks)	4600	80	81–96	2	Lambs from unvaccinated mothers	
	18	Control	–	4600	80	81–96	9		
Sheep 3–4 months	7	rTm16	SC, 3 (2 weeks)	5500	42	120	3	Number of coenuri in rTm18 vaccinated group was not significantly different compared to the controls. Vaccination with rTm16 and/or rTm18 affected the location of coenuri. The number of coenuri detected in the parieto-occipital regions was significantly higher in the controls when compared to the vaccinated sheep. The seroconversion occurred at the time of vaccination. Sheep vaccinated with rTm18 induced a relatively low titer of specific antibody. No significant correlation between the number of parasites detected in vaccinated animals and the titer to rTm16 or rTm18	[45]
	7	rTm18		5500	42	120	3		
	7	rTm16 and rTm18		5500	42	120	3		
	9	Control	–	5500	42	120	9^††^		
Goats 3–4 months old	10	rTm16	^#^SC, 3	5500	^$^14	105	3	Goats vaccinated with coupled rTm16 and rTm18 displayed complete resistance against cerebral coenurosis. Goats in the vaccinated groups developed a few non-cerebral coenuri	[46]
	11	rTm-GST	^#^SC, 3	5500	^$^14	105	2		
	13	rTM16 and rTm-GST	^#^SC, 3	5500	^$^14	105	0		
	10	Control	–	5500	^$^14	105	7		
Lambs 10–12 weeks old	208	Recombinant Tm18	SC, 2 (2–4 weeks)	–	–	–	1	See comment in the text	[47]
	424	Controls	–	–	–	–	32		
Sheep 6 months old	8	Protoscolex cellular metabolite antigen	IM, 2 (3 weeks)	–	–	–	–	The antibody titer (tested by iELISA) increased by 3.4–9.9 times after 1st dose, and by 6.3–12 after 2nd dose. No changes occurred in sera of the controls	[66]
	8	Control	–	–	–	–	–		
Lambs 2–3 months old	16	2-day-old culture of *T. multiceps*	2 (10 days)	Nat	30	NS	4	–	[67]
	16	Control	–				16		
Sheep	6	R45M	4 (3 weeks)	5000	^$^105	14	NS	Vaccinated group had 68.9% reduction in the coenuri numbers compared to controls. Levels of IgG in sera of immune group were significantly higher than those of control	[68]
	6	Control	–	5000	^$^105	14	NS		

*SC* subcutaneous, *IM* intramuscular, *TmAO* *Taenia multiceps* active oncospheres, *ThAO* *Taenia hydatigena* active oncospheres, *Cf* coenurus fluid, *OA* oncosphere antigen, *OSA* oncosphere secretory antigen, *FD-OSA* freeze-dried oncosphere secretory antigen, *NS* not stated, *Nat* turnout on infected pasture

*No. of animals with cerebral coenuri (mature or immature) and/or brain lesions after challenge

^#^Three doses, 2nd dose 1 month after 1st dose. Third dose 7 months after 1st dose

^$^After last vaccination

^†^A single lamb had a live coenurus, and the remaining three lambs had dead (sterile) coenuri

^††^Including five that died because of acute or chronic coenurosis, whereas none of the vaccinated animals had died

However, *Escherichia coli*-expressed recombinant antigens have been successfully used as vaccine candidates against various parasitic diseases [43]. For example, the EG95 recombinant protein vaccine can produce 98% protective immunity against cystic echinococcosis in sheep [44]. This type of vaccine is advantageous since it can be produced on a large scale and can develop a stronger and more specific immune response. Experimental trials to use recombinant antigen vaccines to control coenurosis were first established by Gauci et al. [45]. The authors used homologs of 16k and 18k (Tm16 and Tm18, respectively) families of oncosphere antigens. Although no deaths occurred in the vaccinated group, around 40% of animals of this group had coenuri in their brains after necropsy. Contrarily, all unvaccinated controls displayed cerebral coenuri following the experimental challenge, and > 50% of them had died. This points to a significant level of protection against larvae of *T. multiceps* when using the recombinant Tm16-Tm18 oncosphere antigens; however, this level of protection is lower than that detected when these recombinant antigens were used in other taeniid cestode vaccines, and the authors attributed this to the relatively lower level of antibody response produced against the Tm18 antigen. Contrarily, when Guo et al. [46] used both Tm16 and Tm-GST recombinant antigens, no cerebral coenuri were detected in the vaccinated goats, which suggests development of complete resistance; see Table 4.

Varcasia et al. [47], on the other hand, conducted a successful field trial to control coenurosis in a hyperendemic region (Sardinia, Italy). Six farms were selected with 11.3% mean coenurosis-related mortality (CRM). From these farms, 632 10–12-week-old lambs were selected for the trial. Of them, 208 lambs received a few subcutaneous doses of a vaccine containing Tm18 recombinant protein with 1-month intervals. The remaining 424 lambs served as controls. Over the next 40 months, the mean CRM declined to 5.2%, and no cases of acute coenurosis were observed. Chronic coenurosis was confirmed in a single vaccinated animal, whereas disease was detected in 32 of the controls. In addition, 98% of the vaccinated animals that were serologically tested (*n* = 60) produced specific serum antibodies against the vaccine. Gauci et al. [45], in their experiment, detected a lower antibody response in vaccinated sheep with the Tm18 antigen. A possible explanation for this variation is the difference in strain used. Differences in the nature of the Tm18 protein from various *T. multiceps* strains probably contribute to variabilities in the antigenic characters between these strains. However, nothing is known on variabilities in the Tm18 nature among various *T. multiceps* strains.

## Conclusions

Given that no commercial vaccines are currently available against *T. multiceps* coenurosis, the preventive measures remain the keystone in controlling this disease. These measures include routine anthelmintic dosing of farm dogs with an effective taeniacide, e.g. praziquantel at 5 mg/kg body weight, and controlling the population of stray dogs. Besides, proper disposal of carcasses of the intermediate hosts breaks the transmission cycle of *T. multiceps* by preventing herding and stray dogs as well as wild canids (e.g. foxes) from accessing the coenuri. In addition, increasing knowledge and skills of farmers regarding the cause, clinical signs and prevention of coenurosis is an important strategy to control this economically important disease.

## Data Availability

No datasets were generated or analysed during the current study.
